# HIV Self-Testing Can Be Liberating to HIV-Positive Women and Their Sexual Partners: A Qualitative Study in Kisumu, Western Kenya

**DOI:** 10.1177/2325958220919230

**Published:** 2020-04-29

**Authors:** Kawango Agot, Gift-Noelle Wango, Beatrice Obonyo, Harsha Thirumurthy

**Affiliations:** 1 Impact Research and Development Organization, Kisumu, Kenya; 2 Nyanza Initiative for Girls Education and Empowerment, Kisumu, Kenya; 3 Perelman School of Medicine, University of Pennsylvania, Philadelphia, PA, USA

**Keywords:** HIV self-testing, secondary distribution, HIV-positive women, sexual partners, Kenya

## Abstract

**Background:**

Nearly half of Kenyan men with HIV-positive partners do not know their partner’s status. We carried out a qualitative substudy to explore the experiences of a sample of HIV-positive women when distributing HIV self-tests (HIVST) to their sexual partners.

**Methods:**

HIV-positive women were invited for in-depth interviews to share their experiences in offering HIVST to their partners and how self-testing impacted their relationships.

**Results:**

Two hundred ninety-seven women were randomized to HIVST, 12 of whom self-reported being HIV positive and 11 participated in the interview. Self-testing procedures and interpretation of results were well understood. Participants were strategic in approaching their partners, thus avoided partner violence. Couple testing was high, which strengthened relationships, improved condom use, and empowered women to make joint decisions concerning their health.

**Conclusions:**

Giving HIV-positive women HIVST kits to distribute to their male partners is feasible and safe. Providers who have challenges reaching male partners with testing should consider HIVST.

**What Do We Already Know about This Topic?**

Many studies—including with female sex workers, women attending antenatal and postpartum clinics, and female nurses—have demonstrated that issuing women with multiple HIV self-tests (HIVST) to give to their sexual partners and encourage them to test themselves alone or together as a couple, is acceptable, safe, and effective.

**How Does Your Research Contribute to the Field?**

Although previous studies on secondary distribution of HIVST have focused on HIV-negative women who may find it relatively easy to introduce self-testing to their partners without fearing possible violence, our study explored the experiences of HIV-positive women when introducing HIVST to their partners and provides important strategies that can be adopted by providers in counseling women on how to safely negotiate self-testing with their partners to promote partner and couple testing.

**What Are Your Research’s Implications toward Theory, Practice, or Policy?**

As most African men do not typically visit health facilities for preventive services, our study has added to the utility of HIVST by demonstrating that HIV-positive women can also safely distribute self-test kits to their male partners; hence, women’s HIV-positive status should not bar providers from using this powerful strategy to reach men.

## Introduction

Achieving higher uptake of HIV testing services among high-risk persons is an essential objective of HIV prevention efforts globally and features prominently in the UNAIDS 90-90-90 targets.^1^ Data from Kenya show that men are less likely to use HIV testing services than women, and moreover, only one-third of men and women reported having tested with their sexual partner.^2^ Awareness of HIV status among those who were HIV positive also remained below 90%, particularly for men, and nearly half of men whose partners were HIV positive did not know their partner’s status.^2^ Promoting HIV testing uptake among men is thus an important priority, and this in turn requires consideration of HIV testing modalities that address common barriers to testing that are reported by men. In this regard, HIV self-testing (HIVST) is an important policy option that a number of countries including Kenya have begun to scale-up.

Multiple studies conducted in Kenya, Malawi, and Uganda have now reported that providing women multiple HIVST to distribute to their partners is a safe and effective way to promote partner and couples testing.^3-8^ Women participating in these studies have included health-care workers,^4^ women attending antenatal and postpartum clinics, and female sex workers attending safe spaces.^5,6^ In one study,^6^ 75% to 91% of the women in 3 study settings (antenatal clinic, postpartum clinic, safe space) distributed self-tests to their primary partners, and in majority of these cases, the woman and her partner tested together and learned each other’s HIV status. A subsequent randomized controlled trial comparing the rate of partner and couples testing between women given self-tests versus partner invitations for clinic-based testing showed that self-test provision increased partner testing by 39% and couple testing by 42%.^5^

Despite the promising evidence about secondary distribution of self-tests, the vast majority of studies conducted to date have enrolled HIV-negative women^6^ or men who have sex with men.^3^ Few studies have examined what would happen if HIV-positive individuals were given multiple self-tests and encouraged to initiate partner or couples testing—a strategy that presents greater risks but also potential benefits. Disclosure of HIV-positive status if the woman is the one infected has been associated with gender-based violence,^9-11^ but other studies report that disclosure has in fact led to strengthening of relationships,^12^ including safe sex practices for couples living in serodiscordant partnerships and perhaps an increased likelihood that an HIV-negative partner would seek pre-exposure prophylaxis.^13^

Using data from a randomized trial in Kenya that included a small sample of self-disclosed HIV-positive women, we carried out a qualitative substudy to explore the experiences of these women when distributing HIVST kits to their sexual partners, how their partners reacted to self-testing and the test results, and how their sexual and other relationships were affected by the test results.

## Methods

Data for the substudy were collected in February and March 2016 from participants in a randomized controlled trial (NCT02386215) to promote partner and couple HIV testing through secondary distribution of oral fluid-based self-tests.^5^ The design and methods for the randomized trial have been described elsewhere.^5^ In brief, this trial recruited women seeking antenatal or postnatal services at 3 health facilities in Kisumu County of Western Kenya. Eligible women were aged 18 to 39 years and had a primary partner with unknown HIV status. Those meeting eligibility criteria were consented, administered a structured baseline questionnaire, and randomized 1:1 to an HIVST group or to a comparison group. Those randomized to the HIVST groups received a demonstration of how to use oral fluid-based HIV tests using the manufacturer’s instruction sheet and asked to demonstrate back to ensure that they understood test usage. They were then given 2 OraQuick Rapid HIV-1/2 Antibody Tests (OraSure Technologies, Bethlehem, Pennsylvania) and encouraged to give one to their primary sexual partner and to use one if the partner agreed to couple testing. Importantly, women were told to use their own discretion when determining whether to offer a test kit to their partner and reminded that they were not obligated to offer one to their partner if they felt uncomfortable doing so or if they felt there was a risk of adverse reaction from the partner. They were also asked to inform their partners that even though the self-test kits are highly accurate, it was important to have their test results confirmed at a facility conducting HIV testing, whether the results were negative or positive.

The baseline questionnaire administered to participants inquired about their self-reported HIV status. Follow-up was done 3 months post-enrollment and after the follow-up period ended, participants who reported being HIV positive in the HIVST group were contacted for in-depth interviews. Trained research assistants used a guide to explore how participants used the 2 test kits, experiences of offering the kits to their partners, reaction of the partners to HIVST and to the test results, and if introducing HIVST kit and testing affected their relationships (see online supplemental materials). The interviews were conducted in English, Kiswahili, or Dholuo, audio-recorded, and subsequently transcribed. Two of the authors each prepared a code book, with key codes covering the themes in the interview guide: testing procedures, reasons for offering test kits to partners, strategies used to offer the kits, reaction of partners to the kits and to the results, and how introducing self-tests affected the relationships. The codes were compared and synchronized, then used to code the rest of the transcripts, which were reviewed with the lead author to ensure salient themes were captured and suitable quotes selected for inclusion.

### Ethical Approval and Informed Consent

Ethical approvals were obtained from the Kenya Medical Research Institute’s Scientific and Ethics Review Unit (Ref: Non-SSC Protocol No.471) and the Office of Human Research Ethics of the University of North Carolina at Chapel Hill (Ref #14-3040). All participants were administered a written informed consent in their preferred language (English, Kiswahili, or Dholuo) by trained trilingual study staff who had received ethical training on research with human participants. Participants gave consent to participate in the main study and if selected, in the qualitative substudy that included optional audio-recording of the interviews.

## Results

A total of 600 participants were enrolled in the trial and 297 were randomized to the HIVST group. Twelve participants from the HIVST group self-reported being HIV positive and 11 agreed to participate in the qualitative substudy and one had relocated outside the study area. Key characteristics and behavioral outcomes of these participants are summarized in Table 1. Among the 8 participants who had previously disclosed their HIV status to their partners, 5 were using condoms consistently both before and after the self-test, while one became a consistent user and another started using condoms after testing; one partner declined HIVST and had stopped having sex when the participant disclosed her status to him earlier. Five tested together as couples while 2 partners tested on their own. Only 3 women had not disclosed their status to their partners, of whom 2 started consistent condom use after the test and 2 confirmed their test results. Couple and partner testing were comparable between those who disclosed and those who did not.

**Table 1.** Characteristics of Study Participants and Key Outcomes.

Pseudonym	Age (Years)	Highest Education Level	Partner Result	Confirmed Results	Previously Disclosed Status to Partner	Tested Together	Condom Use Following HIVST
Amondi	26	Primary	Negative	No	Yes	Partner testing	Had been using condoms consistently; continued after HIVST
Akinyi	26	Some secondary	Negative	No	Yes	Couple testing	Had been using condoms consistently; continued after HIVST
Anyango	28	Some secondary	Positive	Yes	No	Partner testing	Not having sex even before HIVST because partner declined to use condoms
Achieng	25	Secondary	Negative	No	Yes	Couple testing	Condom use was inconsistent; became consistent after HIVST
Adhiambo	26	Secondary	Negative	Yes	Yes	Couple testing	Not using condoms previously; started using after HIVST
Atieno	37	Primary	Negative	Yes	No	Couple testing	Not using condoms previously; started using after HIVST
Awuor	27	Secondary	Negative	Yes	Yes	Couple testing	Had been using condoms consistently; continued after HIVST
Awino	19	Some secondary	Negative	Yes	Yes	Couple testing	Had been using condoms consistently; continued after HIVST
Auma	26	Postsecondary	Negative	Yes	Yes	Partner testing	Had been using condoms consistently; continued after HIVST
Amollo	23	Postsecondary	Did not test	NA	No, but sees me taking drugs daily	Participant self- tested alone	Condom use was inconsistent; became consistent after reconfirming her status using HIVST.
Akumu	27	Some secondary	Did not test	NA	Yes	None tested	Not having sex; man changed sleeping room when learnt partner was positive

Abbreviations: HIVST, HIV self-test; NA, not applicable.

Of the 9 partners who tested, 8 were negative. Even though the participant with a positive partner had not disclosed her status to him, the couple had stopped having sex because the man refused to use a condom. The negative partners mostly tested together as a couple (n = 6), had disclosed their HIV status to their partners (n = 7), and were consistent condom users both before and after HIVST (n = 5); the remaining 3 became consistent users after the test. A participant who self-tested when the partner declined testing became consistent user after confirming her own HIV status. Among the 6 who tested as a couple, there was no clear relationship between couple- or partner-testing and condom use, previous disclosure of HIV status to the partner, or confirmation of test results.

Of the 11 participants, 9 reported that their partner tested and 2 partners declined testing. Six participants reported using self-tests together with their partner, some in order to encourage them to test or to confirm their own status hoping the results could change to being negative because the testing approach was different: “I was just hoping that a miracle could happen…my heart was beating fast, I thought it may show me a negative result…I was just saying that the other one was tested using blood but this one I have used saliva maybe this thing can be different” (Atieno, 37 years; ages of all participants actual but names changed to protect confidentiality). Others felt there was no need for testing with their partners since they already knew their status. However, only 8 had disclosed their HIV-positive status to their partners and 6 of the 9 whose partners tested went for confirmatory testing, with all results being confirmed accurate. Below, we present participants’ experiences when offering HIVST to the partners, how partners who self-tested reacted to self-testing and to the results, and if self-testing affected sexual and other relationships.

### Participants’ Understanding of Self-Testing Procedures

Although the in-depth interviews were conducted 4 to 8 months after self-testing procedures were first demonstrated to participants, explaining testing procedures to partners and interpreting the test results seemed to have been understood relatively well. Some used the instruction sheets provided:

I brought out the test kit, put it on the table then I told him “now we want to follow the instruction one by one.”…Then I told him how to use them, how you [Research Assistant] explained. Right now, I have forgotten some things but by that time I knew because I was reading and remembering what you had told me. (Achieng, 25)

Others did not mention relying on the written instructions:

I just told him how that thing can be used…inside the test kit there was something you take then you swab on the gums like this [RA: participant demonstrates], so it was both sides so you swipe one side on the lower gum and again you turn and swipe your upper gum. Then there is, there is something that had some liquid where you would insert it after doing that, then you wait for the results to come out. After the results are out, if it crosses two lines then that one you are positive, if it crosses one line then you are negative. (Auma, 26)

Participants mostly understood the time required for the test to run as well as the time between the last meal or drink before being tested:

…then 15 minutes before he ate anything, he swabbed up and down his mouth then he inserted in the bottle of…that has liquid, then he waited for 20 minutes (Amondi, 26) or that “It is used before eating and if you test after eating let 15 minutes elapse before you test.(Awino, 19)

None of the participants mentioned the 40-minute upper limit, with one warning that waiting beyond 20 minutes may yield a different result.

However, maybe because of passage of time between the time the procedures were demonstrated and when participants were interviewed (4-8 months), some were not as clear about the length of time required to abstain from eating or drinking before conducting the test, or conflated it with the time recommended after a mouth wash, which is 30 minutes:

The way it was explained to me was that I should use them something like 30 minutes before I eat something. I took my time, got into the house and took 30 minutes, did not eat or use anything for that period. (Amollo, 23)

### Why Participants Offered Self-Tests to Their Partners

Participants were clear on the reasons for offering self-test kits to their partners, with majority reporting being motivated by the desire to have their partners know their own status and if positive to start taking antiretroviral medication. For others, it was to help them practice safe sex “since we have sex and I am positive,” or more elaborately:

…with my husband I told him it (testing) will help us participate in what is called safe sex and see how we can manage our family. [RA probe: what do you mean by safe sex?]. Safe sex is using condoms when having sex, safe sex is communicating to one another that agreement…if you don’t agree you can’t practice safe sex…it is to be faithful, use condoms…(Achieng, 25)

Others used the convenience and confidentiality of self-testing to get their partners to test:

I tried to explain to him that “some (people) find it difficult but being that this new research helps those who would like to know their status, but because going where they can be seen make them fear, this one for home helps someone to know his status easily…” There was a time we tried to go, he had the will of going with me but when he reached there [the facility] he saw several people he knew and he turned back. (Awuor, 27)

### Participants Strategic in Introducing HIVST to Their Partners

It was clear that participants understood their partners well and consciously decided on the most opportune time and place to introduce self-testing. They reported waiting until “the children had slept and we were alone,” or “when we were relaxed and watching programs [TV].” The majority emphasized the need for politeness and to know when to engage their partners and when to disengage. Several women hid behind the study and the new testing strategy so as not to appear as if the idea of bringing the self-test kits home was premeditated:

He asked me “where did this come from.” I told him “when I took the baby to the clinic I was referred to some researchers. I was asked questions which I answered and accepted that I would enter (join) the study…I was told how to enter, how I can test myself with them (self-test kits), how long to wait and see two lines on the kit, one line means there is no virus and if it has two lines there is HIV.” Then he asked how that thing is used then I told him. He did that (tested) at night. (Anyango, 28)

A participant challenged her partner on the need to test together as a couple:

We sat together as a couple in the house then after taking our supper we talked. At first, he said that he went (for testing) two months ago. I was not convinced, yes, I told him that “I know you should be going for that test after every three months but I wasn’t around, now it is time for you to get tested together with me…” I told him about the self-test kits, then he agreed after so many questions about how HIV can be in the saliva but at the end we decided to be tested because he reasoned that, “I am living with a positive person, now I should do what, be tested.” (Achieng, 25)

Other participants used the convenience of the process of self-testing and confidentiality of the results to convince their partners to test, reminding them how busy they are to get time to go to hospital and how confidential self-testing is. For another, once the partner agreed to test, she opted for a shared, more intimate, approach in which each read the results of the other:

He accepted then first read the paper [instruction sheet] I was given…I then put the stands down, mine in front of him and I put his in front of me. He wanted us to put them at ago, so when we put saliva I put mine and he put his and we dipped inside that thing and let them stand after dipping. I was just waiting for his results, how it was going to come out because his results were the ones in front of me, mine was in front of him. (Adhiambo, 26)

A participant shared a creative way of convincing her partner to test and used the opportunity to disclose her own status to him:

I was very grateful for those things [self-tests]. I have always tried to ask him to go and he refused saying those are trips he has no time for…(So) I first cheated him and put both of them on the table. I removed his and told him to test first then I will test…When he finished testing and the results were ready and he saw his, he sat down and kept quiet then asked me why I was not testing. I removed my clinic card and showed him and told him, “I had tested, you were the only one remaining.” He did not know I was on care…that is the day he knew (Anyango, 28)

Two participants, however, were unsuccessful in convincing their partners to self-test, even with equally innovative strategies:

I had told him that “today I have brought for you something good”…when he looked inside he found that it was a paper he threw it away…I told him, “why don’t you first read what is inside it to get what it is all about or if you can sit, then sit I read for you as you hear.” He told me that his mind was so full that he could not even read. I told him “I can read for you” then he told me that he was so tired he does not want to hear what is read to him. As I started reading…my eyes were on the paper not knowing that I was reading to someone who was already asleep…My thought, by my own thoughts, I think that could be he has HIV (Akumu, 27)

### Reaction of Participants and Their Partners to Self-Test and Test Results

We explored what participants and their partners thought about HIVST and the test results. Most participants found both liberating in 4 important aspects: it offered them an opportunity to know the status of their partners and for the partners to know their own status hence a relief, it provided participants an opportunity to disclose their status to their partners, it offered couples in discordant relationships an opportunity to act on their status, and it led to improved partner support and safer sex practices.

#### HIV self-test provided an opportunity of knowing each other’s HIV status

To all participants, knowing each other’s status as a couple was important in relieving uncertainty about their lives individually, as a couple and as a family:

I think that if I wouldn’t have brought the test kits home, did it together and the result turned out to be negative for him and positive for me, and if it would have turned out to be positive for him I think it could have been worse on my side and everything which was being done for me would have stopped. He would have blamed me for that (infecting him)…What I usually pray to God to relieve me of is that let him stay like the way he is (negative) and let me stay the way I am so that there should be no blame between us (Achieng, 25)

To others, it provided an opportunity to confirm and accept their own status:

That thing (test kit) confirmed to me that I have HIV, and it found that I had been tested once so it was like I repeated the test. So that thing confirmed to me that it is something real, that is, the rate of accepting (my positive status) became higher. (Amollo, 23)

#### HIV self-test improved relationships

To the majority, the relationship improved with testing and knowing each other’s status. There were those who were initially uncertain about how their partners would react but all ended well:

Before I was feeling my heart troubled. I had some fear because he hadn’t seen what I was telling him…you know if someone hasn’t seen your status you’re just telling him it is different from when he sees it…so I was thinking maybe he could have some reactions when he sees for himself that it’s true and that was making my heart get troubled…right now (after testing) when I’m taking medication he can remind me, even when I am giving the child medicine he can remind me. (Atieno, 37)

Although for another group, HIVST made the relationship more solid.

The support has increased as he reminds me to take my drugs all the time…he tells me how I should take care of myself and he ensures that I feed well…even if I was busy and it is about the time I should be taking my drugs he reminds me…and he reminds me of the day I should be going back to the clinic. (Amondi, 26)

It (our relationship) came out to be much stronger, the happiness and the bond, because from that time on we’ve been usually…we were a happy family, yes, but there is some kind of situation where being together, talking together as husband and wife was difficult but from that time…we usually sit down, talk, discuss and see the way forward on how to manage our family, to take care of our family. (Achieng, 25)

Some participants were confident that their HIV status would not come in the way of the love they had for one another:

In his mind when I studied him, I saw he was down, but later I encouraged him and told him that we just continue the way we were because that’s how he had known me…Later he encouraged me that I’m not the only one who was that way, that so many people are that way…that I should not, I should not give up in life. These days he often takes me to the clinic. (Awuor, 27)

Testing exonerated some partners from the guilt of feeling they were the source of infection to their wives:

When I told him (my HIV status) it’s like he thought because we have never gone (for testing) together sometimes I can think he is the one who infected me because he made me pregnant…so he was eager for me to see his status. (Atieno, 37)

Anyango, 28, was also glad her partner self-tested and linked to care:

Yes the following day in the morning as he was going to work, he went alone…he came and told me that they just found it just like it was (positive)…He then went to [facility name redacted] for care and was just put on ARVs direct because his CD4 was too low.

#### HIV self-test improved condom use

We explored if knowledge of test results led to changes in sexual relationships, specifically in point-of-sex decision-making. One participant reported not having sex since after delivery because the partner refused to use condoms so she withheld sex from him; 5 reported continuing with consistent condom use because they had disclosed their HIV status to their partner earlier; and one tested alone to reconfirm her status, then decided to use condoms consistently:

I was at times using condoms and sometimes I was not using…so after I used the test kit, it’s like it confirmed to me that this thing (HIV) is truly there, so it made me not to miss using condoms. (RA: Why were you previously not using condoms sometimes yet you knew you were positive?): You know, the reason why that one was happening I had not accepted [the positive result] or I had not accepted in my heart, it was still disturbing me, I was saying that maybe the machines are lying to me, yes, that is what was happening.

For 4 participants, self-testing marked the start or consistency of condoms use. Because they had not disclosed their status to their partners, they were unable to insist on condom use. One explained: “Now the reason why we use condoms after we had tested using the test kit you gave us is when he saw my results…we now use condoms all the time” (Adhiambo, 26) while others were more elaborate:

Previously I used to tell him (to use condoms), he used to say he had no problem and if I was thinking he had a problem then we could go and be tested and I used to ask myself what if I tell him my status…maybe he could feel bad and think that I know my status and it’s like I want to harm him (infect him)…So that could make us to sometimes use or not use but after getting to know each other’s status, we agreed and decided that if we are staying (together) we have to stay with that (condoms)…now when sometimes he insists on not using I usually tell him that I like, I want him to remain like he is (negative) (Atieno, 37)

There is one thing which changed because with my husband, let me just be honest…there were times he wasn’t using condoms…but ever since, he hasn’t stopped using that condom ever since we tested together…because he knew that if you don’t talk together, come to terms with what we were (serodiscordant), you can contaminate…you can contact HIV because if you force yourself inside without using condoms, it is a chance of 50/50 for you to contact the virus (Achieng, 25)

## Discussion

In this qualitative study among HIV-positive women who were given multiple self-tests, we found that despite their HIV-positive status, they were able to safely offer a self-test to their primary sexual partner and initiate partner or couples testing. Women were strategic in approaching their partners by carefully thinking through when and where to bring up the subject and what to say, which made them avoid confrontation. When they sensed hesitation from their partners or because they knew that their partners were generally difficult, they introduced the discussion indirectly, often using the study or “doctor” at the maternal and child health clinic as the reason they brought home the self-test kit, thus avoiding being perceived by their partners as having a hidden agenda in coming home with the kits. They appealed to their partners by reminding them of the convenience and confidentiality of HIVST vis-à-vis clinic- or provider-supported testing and used the partner’s busy schedules or stigma associated with clinic-based testing to convince their partners to consider self-testing. Importantly, all women were able to offer HIVST to their partners, attesting to the intrinsic boldness women can exhibit for a cause they firmly believe in—in this case, the desire to have their partners know their HIV status and make decisions about their own health moving forward, and for both to chart their life paths as a family with the knowledge of each other’s status.

Participants reported that demonstrating self-testing procedures to their partners was easy. Although our participants may not have remembered every detail with the precision of laboratory technologists, they knew the minimum skills required by a layperson to successfully conduct the test, specifically the requirement of not eating or drinking before conducting the test, how and where to swab, how long to wait, and how to interpret the results. A study among female health-care workers in Kenya also reported that using the self-test kit was easy, even with no supportive video demonstration or written instructions or leaflets to bolster their skills.^4^ Similar findings are reported in other studies in Kenya, with participants also reporting that using self-test kits was easy.^14,15^ In Malawi, Choko and colleagues^16^ found very high accuracy among lay users after a brief demonstration with illustrated instructions and reported that just 10% of participants, especially among those with no or low literacy, made minor procedural errors. On the other hand, in a multisite study in Kenya, Malawi, and South Africa, in which participants were simply given instructions to read and follow, with no demonstration (akin to what would happen if they were to buy the self-test kit off a commercial outlet) and videotaped on how they conducted the test reported that participants missed various steps, including shortening waiting time and using the wrong end of the swab; however, the authors concluded that participants were, by and large, able to interpret the results correctly except where the lines were weak or results invalid.^17^

Our findings support studies conducted among other population groups that also reported no or minimal social harm experienced as a result of sharing the test results with a partner. Such studies enrolled sex workers, antenatal and postnatal women who offered self-testing to or tested together with their partners,^5,6^ men who have sex with men who offered self-testing to or tested together with their partners,^3,18^ women in the general population who tested with their partners,^16^ or cohabiting couples.^19^ We have demonstrated that HIV-positive women in our study understood their partners well enough to navigate through the process effectively and safely. However, the fact that most women had already disclosed their positive status to their partners prior to bringing HIVST home may have contributed to the safe distribution of the kits. Similarly, Ngure and colleagues^14^ reported no social harm following HIVST by HIV-negative partners in a discordant couple relationship which the authors partially attributed to couples’ prior knowledge of each other’s status. The fact that most women in our study, despite being HIV positive, tested together with their partners—almost all of whom were HIV negative—and no violence ensued, is a demonstration that women may be more empowered in making decisions about their own and their family’s health than is presented in literature.

Women in our study found HIVST empowering. They reported that the support their partners gave them after testing allayed their fear of rejection or violence on account of being positive and enhanced their compliance with elements of positive living, specifically condom use and adherence to medication and clinic visits. This finding is consistent with results from several studies: participants in serodiscordant relationships who were on Pre-Exposure Prophylaxis (PrEP) found HIVST empowering by reducing the anxiety associated with going to the clinic for retesting before PrEP refill.^14^ Both male and female participants in a Malawi study^19^ found HIVST to be empowering in that mutual knowledge of each other’s HIV status cultivated openness and psychosocial support among partners, including support to adhere to ART and disclosure. On the other hand, HIV-negative female sex workers in Kenya whose partners tested positive reported mixed reactions—including experiencing verbal abuse and terminating sexual relationships with the positive partner.^20^

A major weakness of this study is the small number of participants interviewed. However, the views expressed covered a complete spectrum of issues that HIV-positive women have cited with regard to benefits of disclosure of status, such as improvement in practicing safe sex and supporting adherence and clinic visits.^12,13,20^ In addition, the experiences of our participants reflected a wide range of issues reported by HIV-negative women who were also given HIV test kits to distribute to their partners.^14,19,20^ We are therefore confident that despite the small sample size, our results can apply to other HIV-positive women who may consider testing with their partners using self-test kits. We also did not collect information on the age of partners or length of relationships, thus unable to assess how power dynamics might have played out following self-testing especially in age disparate or short- versus long-term unions. Importantly, we did not interview the partners to verify the information obtained from the participants. Finally, there is the possibility that the time lag between the distribution of the self-test and the interview (4-8 months) may have affected participants’ ability to clearly recall details of condom negotiation or partner reaction to HIVST and to the test results.

The study had several strengths. To our knowledge, this is one of the first studies to qualitatively explore experiences of HIV-positive women in distributing oral HIVST to their partners; in addition, it provided an opportunity for couples who were unknowingly living in a discordant relationship to know each other’s status and take action to remain negative or link to care, as appropriate. The Malawi study^19^ also found that 7 of the 9 HIV-infected HIVST users were in a discordant relationship which they were unaware of previously, comparable to the proportion we found in in our study.

In conclusion, HIV-positive women in the Kenyan study setting were able to safely distribute HIVST kits to their sexual partners, discuss with them the importance of getting tested, and decide to have protected sex. Providing multiple self-tests to HIV-positive individuals with the objective of promoting disclosure of HIV status, partner testing, and couples testing is thus a strategy that warrants greater consideration by HIV programs, health-care providers, and policymakers. Coupled with studies that have demonstrated that secondary distribution of self-tests by HIV-uninfected women is an effective way to promote partner and couple testing, this study adds to this discussion by demonstrating that HIV-positive women are equally capable of safely offering self-test kits to their partners.

## Supplemental Material

Supplemental Material, HIV-positive-women-and-HIVST-Manuscript_ID-JIAPAC-19-07-OM-1176_Supplementary-Material_IDI-Guide - HIV Self-Testing Can Be Liberating to HIV-Positive Women and Their Sexual Partners: A Qualitative Study in Kisumu, Western Kenya Click here for additional data file. Supplemental Material, HIV-positive-women-and-HIVST-Manuscript_ID-JIAPAC-19-07-OM-1176_Supplementary-Material_IDI-Guide for HIV Self-Testing Can Be Liberating to HIV-Positive Women and Their Sexual Partners: A Qualitative Study in Kisumu, Western Kenya by Kawango Agot, Gift-Noelle Wango, Beatrice Obonyo and Harsha Thirumurthy in Journal of the International Association of Providers of AIDS Care (JIAPAC)

Supplemental Material, HIV-positive-women-and-HIVST-Manuscript_ID-JIAPAC-19-07-OM-1176_Supplementary-Material_Questionnaire - HIV Self-Testing Can Be Liberating to HIV-Positive Women and Their Sexual Partners: A Qualitative Study in Kisumu, Western Kenya Click here for additional data file. Supplemental Material, HIV-positive-women-and-HIVST-Manuscript_ID-JIAPAC-19-07-OM-1176_Supplementary-Material_Questionnaire for HIV Self-Testing Can Be Liberating to HIV-Positive Women and Their Sexual Partners: A Qualitative Study in Kisumu, Western Kenya by Kawango Agot, Gift-Noelle Wango, Beatrice Obonyo and Harsha Thirumurthy in Journal of the International Association of Providers of AIDS Care (JIAPAC)
