# Supplementary material for: HIV Self-Testing Can Be Liberating to HIV-Positive Women and Their Sexual Partners: A Qualitative Study in Kisumu, Western Kenya
Source: J Int Assoc Provid AIDS Care. 2020 Apr 29;19:2325958220919230. doi: 10.1177/2325958220919230 (PMC7236088; doi:10.1177/2325958220919230)
Supplement: Supplemental Material, HIV-positive-women-and-HIVST-Manuscript_ID-JIAPAC-19-07-OM-1176_Supplementary-Material_IDI-Guide - HIV Self-Testing Can Be Liberating to HIV-Positive Women and Their Sexual Partners: A Qualitative Study in Kisumu, Western Kenya [file HIV-positive-women-and-HIVST-Manuscript_ID-JIAPAC-19-07-OM-1176_Supplementary-Material_IDI-Guide.pdf]

The use of HIV self-tests to promote partner and couples testing: a randomized trial

In-depth Interview Guide

Participants:

- Women enrolled in the intervention arm of the study who report using the HIV self-test with their partner as a couple
- Women enrolled in the intervention arm of the study who report their partner used the HIV self-test independently
- Women enrolled in the intervention arm of the study who report that they did not use the HIV self-test with partner/partners

Topics covered:

- How women discussed HIV self-testing with their partners
- The experience of using the HIV self-test with a sexual partner
- Why women did and did not use the HIV self-test
- How using the HIV self-test affected women's decisions about sexual behaviors and health behaviors after using the self-test
- The reactions of sexual partners to HIV self-testing and reports of any violence or other negative experiences

Interviewer Introduction:
Thank you for talking to us. When you agreed to participate in the "HIV self-testing study" you agreed to be contacted for an in-depth interview. This interview will help us find out more about your experience in the study and using the HIV self-test. There is no right or wrong answers to these questions. Please be as honest as possible; your answers will help us improve HIV self-testing programs in the future. You can skip any questions you do not want to answer and you can end this interview at any time. Do you agree to participate in this interview? [If woman says no, then thank her and end the interview.]

In order to help me remember what we discuss today, I would like to audio record our discussion. The recording can be stopped at any time and will be destroyed once the study is completed. Do you agree to be recorded? [If woman says no, you may continue and take notes on paper].

☐ If participant agreed to be audio recorded

☐If participant declined to be audio recorded

Are you ready to start?

Recruitment Site:____

Participant Age:______________ Participant Marital Status:____________________

IDI Group: (circle one) Tested as couple / Partner tested individually / Partner did not test

Date:____

Introductory questions (Mandatory for all participants).

1. Tell me about your family. Who do you live with? (*Probe for marital/relationship status, family dynamic, number and age of children*)

2. Before this study, what had you heard about HIV self-testing? Where did you hear this? When did you hear about this? What did you hear? From whom?*(ensure you capture the source and confirm that they are not talking about the standard H.T.C)*

3. After becoming enrolled in the study, did you discuss HIV testing or using the self-test with your partner? Could you tell me about what you discussed?

4. Please share with us what happened to the two test kits you were given *(probe whether both were offered and whether both were used)*

5. Did you and/or your partner use the HIV self-test?
    a. Please explain who used the test and whether you used it together or separately.

Questions for women who used the self-test together with their partner (Couples Testing):

(Next we are going to ask you a set of questions about your experience giving out and/or using the self-test kit.)

6. Could you tell me about using the HIV self-test with your partner? (*Allow the participant to tell her story. Probe for location, context that prompted her or made it easy for you to offer)* What did you tell him about self-test? How did you talk to him about the self-test? (*Probe for words and/or arguments used, coercion*)
    a. Can you briefly walk me through how you gave him the instructions on how to use the test kit? What questions did he have? How did you respond to the questions, if any?

7. What was your partners' reaction when you brought up the topic of HIV self-testing? What happened next? *(Probe for positive and negative reactions, words used, violence and adverse events)*

8. How did you decide to take the HIV self-test together?

(<u>*Interviewer*</u>*: probe for how soon it was used after she offered it; was there any trigger/reason for his decision to use it at that time?*).

9. What was using the self-test like? Tell me about how you used it?
    a. What did you do while waiting for the results? What did he do while waiting for the results? How long did you wait?
    b. How did you feel while waiting for the results?

10. Tell me about the result. What was your partner's reaction to getting their test result? What happened next?
    a. Did you talk about your results with each other? What was that like?
    b. Was it easy or hard to read the test result? Why?
    c. What was your reaction to his results?
    d. What was his reaction to your results?

11. Did anything change as a result of taking the test? What? Tell me about that
    (Probe for relationship change, e.g. relating to each other as a couple and relating to each other as family; changes in sexual behavior or condom use. Probe whether the changes were as a result of her test results or his)

    a. Did you and your partner have sex after taking the test? Did you use condoms?
    b. Did it change anything in your relationship?
    c. Did knowing each other's status through self-testing change your normal relationship (how did it change and why?).

12. Did you get a confirmatory test at the HTC?
    a. Why or why not?
    b. What was that experience like?
    (Probe for participant and partner separately)

13. <u>If positive result</u>, have you or your partner sought care? (*probe for where, what experience was like, if went together or separately*)

14. <u>If not mentioned</u>, did you experience any conflict or violence with your partner around this test? Tell me about that…

<u>Questions for women whose partners used the self-test alone (Partner Testing):</u>

15. Could you tell me about when your partner used the HIV self-test? (*Allow the participant to tell her story. Probe for location, context what prompted her or made it easy for you to offer).*
16. What did you tell him about self-test? How did you talk to him about the self-test? (*Probe for words and/or arguments used, coercion*)

   a. Can you briefly walk me through how you gave him the instructions on how to use the test kit? What questions did he have? How did you respond to the questions if any?

17. What was your partners' reaction when you brought up the topic of HIV self-testing? What happened next? (*Probe for positive and negative reactions, words used, violence and adverse events*)

18. How did you decide to take the HIV self-test separately?
(*<u>Interviewer</u>: probe for how soon it was used after she offered it; was there any trigger/reason for his decision to use it at that time*).

19. What was using the self-test like? Tell me about how he used it?
    a. What did you do while waiting for the results? What did he do while waiting for the results?
    b. How long did you wait?
    c. How did you feel while waiting for the results?

20. Tell me about the result. What was your partner's reaction to getting their test result? What happened next?
    a. Did you talk about your results with each other? What was that like?
    b. was it easy or hard to read the test result? Why?
    c. What was your reaction to his results?
    d. What was his reaction to your results?

21. Did anything change as a result of taking the test? What? Tell me about that. (*Probe for relationship changes, changes in sexual behavior or condom use*)
    a. Did you and your partner have sex after he used the test? Did you use condoms?
    b. Did it change anything in your relationship?
    c. (If you shared your results)Did knowing each other's status through self-testing change your normal relationship (how did it change and why?).

22. Did you get a confirmatory test at the HTC?
    a Why or why not?
    b What was that experience like?

23. <u>If positive result</u>, have you or your partner sought care? (*probe for where, what experience was like, if went together or separately*)

24. <u>If not mentioned</u>, did you experience any conflict or violence with your partner around this test? Tell me about that…

<u>Questions for women who neither used the HIV self-test kits nor their partners:</u>

25. Tell me why you or your partner did not use the HIV self-test. (*Allow the participant to tell her story, probing for additional detail such as fear, partner refusal etc*.)

26. Did you talk to him about HIV testing? What did you tell him about self-test? How did you talk to him about the self-test? (*Probe for words and/or arguments used, coercion*)

27. What was your partners' reaction when you brought up the topic of HIV self-testing? What happened next? (*Probe for positive and negative reactions, words used, violence and adverse events*)

28. How did you decide to not use the HIV self-test?
    d. Was it a decision you made without talking to your partner or did you discuss it together?
    e. Did he refuse to use the self-test? Why?

29. Did anything change as a result of being in this study? What? Tell me about that. (*Probe for relationship changes, changes in sexual behavior or condom use*)
    f. Have you and your partner had sex since joining the study? Did you use condoms?
    g. Did it change anything in your relationship?

30. <u>If not mentioned</u>, did you experience any conflict or violence with your partner as a result of being in this study? Tell me about that…

<u>Closing Questions:</u>

31. After participating in this study, what recommendations do you have about HIV self-tests? What would you tell other people about it? What could we do to improve the HIV self-test?

32. Do you have anything else you'd like to share?

Thank you for your time and for participating in this interview.

Offer resources: for confirmatory test, linkage to care, or IPV at the end of the interview as needed.
