# Supplementary material for: HIV Self-Testing Can Be Liberating to HIV-Positive Women and Their Sexual Partners: A Qualitative Study in Kisumu, Western Kenya
Source: J Int Assoc Provid AIDS Care. 2020 Apr 29;19:2325958220919230. doi: 10.1177/2325958220919230 (PMC7236088; doi:10.1177/2325958220919230)
Supplement: Supplemental Material, HIV-positive-women-and-HIVST-Manuscript_ID-JIAPAC-19-07-OM-1176_Supplementary-Material_Questionnaire - HIV Self-Testing Can Be Liberating to HIV-Positive Women and Their Sexual Partners: A Qualitative Study in Kisumu, Western Kenya [file HIV-positive-women-and-HIVST-Manuscript_ID-JIAPAC-19-07-OM-1176_Supplementary-Material_Questionnaire.pdf]

3ie Self Testing 2 Study
Baseline questionnaire

Section A: Participant information			
NO.	QUESTIONS	CODING CATEGORIES	
1	Recruitment location	1 LHC<br>2 KEDH<br>3 Rabuor	
2	ANC or PPC	1 ANC<br>2 PPC	
3	Interviewer ID		[1 digit]
4	Date of Interview	(DD/MM/YYYY)	
5	Time of interview	(HH:MM)	
6	Participant ID	XX-XXX-XXXX-XXXX<br>Loc-site-MMDD-HHMM	[created by ODK]

Section B. Demographic Information			
NO.	QUESTIONS	CODING CATEGORIES	NOTES
1	What is your age?	NUMBER OF YEARS<br>2 digits	Interviewer: should be consistent w/ screening
2	Have you attended any school?	1 YES<br>2 NO→3	
3	What is the highest level of school you have attended?	1 NONE<br>2 PRIMARY – INCOMPLETE<br>3 PRIMARY – COMPLETE<br>4 SECONDARY-INCOMPLETE<br>5 SECONDARY–COMPLETE<br>6 HIGH SCHOOL<br>7 POST-SECONDARY/HIGH SCHOOL TRAINING COLLEGE<br>8UNIVERSITY<br>98 DON’T KNOW<br>99 REFUSED TO ANSWER	
4	What is your current marital status?	1 MARRIED, LIVING TOGETHER<br>2 MARRIED, NOT LIVING TOGETHER<br>3 NOT MARRIED, LIVING TOGETHER<br>4 DIVORCED/SEPARATED<br>5 WIDOWED<br>6 NEVER MARRIED AND NOT LIVING TOGETHER<br>98 DON’T KNOW<br>99 REFUSED TO ANSWER	
5	Do you have any children?	1 YES<br>2 NO→6	
6	How many living children do you have?	(NUMBER)<br>98 DON’T KNOW<br>99 REFUSED TO ANSWER	
7	If pregnant, what is your due date?	(DD/MM/YYYY)	ANC only
8	When did you give birth?	(DD/MM/YYYY)	PPC only
9	What is your ethnic group/tribe?	1 LUO<br>2 LUHYA<br>3 KALENJIN<br>4 KIKUYU<br>5 KISII<br>6 OTHER: ________________________ (SPECIFY)<br>98 DON’T KNOW<br>99 REFUSE TO ANSWER	

10	What is your primary occupation?	1 PROFESSIONAL<br>2 CLERICAL<br>3 SALES AND SERVICES<br>4 SKILLED MANUAL<br>5 UNSKILLED MANUAL<br>6 DOMESTIC SERVICE<br>7 AGRICULTURE (OWN LAND)<br>8 AGRICULTURE (PLANTATION/OTHER)<br>9 SEX WORK<br>10 UNEMPLOYED →<br>11 STUDENT →<br>12 OTHER__________________________ (SPECIFY)<br>98 DON’T KNOW<br>99 REFUSED TO ANSWER	
11	How much income do you typically earn each month?	KES________#<br>98 DON’T KNOW<br>99 REFUSED TO ANSWER	
12	Who usually decides how the money you earn will be used: mainly you, mainly your husband/partner, or you and your husband/partner jointly?	1 Respondent<br>2 Husband/Partner<br>3 Respondent and Husband/Partner Jointly<br>4 Other (specify___)<br>98 DON’T KNOW<br>99 REFUSED TO ANSWER	
13	Would you say that the money that you earn is more than your husband/partner earns, less than what he earns, or about the same?	1 More than him<br>2 Less than him<br>3 About the same<br>4 Husband/partner does not earn any money<br>5 Other (specify___)<br>98 DON’T KNOW<br>99 REFUSED TO ANSWER	
14	Who usually decides how your husband/partner’s earnings will be used: mainly you, mainly your husband/partner, or you and your husband/partner jointly?	1 Respondent<br>2 Husband/Partner<br>3 Respondent and Husband/Partner Jointly<br>4 Husband/partner has no earnings<br>5 Other (specify___)<br>98 DON’T KNOW<br>99 REFUSED TO ANSWER	
15	Who usually makes the decisions about health care for yourself: you, your husband/partner, you and your husband/partner jointly, or someone else?	1 Respondent<br>2 Husband/Partner<br>3 Respondent and Husband/Partner Jointly<br>4 Someone Else (specify___)<br>98 DON’T KNOW<br>99 REFUSED TO ANSWER	
16	Who usually makes the decisions about making major household purchases: you, your husband/partner, you and your husband/partner jointly, or someone else?	1 Respondent<br>2 Husband/Partner<br>3 Respondent and Husband/Partner Jointly<br>4 Someone else (specify___)<br>98 DON’T KNOW<br>99 REFUSED TO ANSWER	
17	Who usually makes decisions about making purchases for daily household needs? You, your husband/partner, you and your husband/partner jointly, or someone else?	1 Respondent<br>2 Husband/Partner<br>3 Respondent and Husband/Partner Jointly<br>4 Someone else (specify___)<br>98 DON’T KNOW<br>99 REFUSED TO ANSWER	
18	Who usually makes decisions about visits to your family or relatives? You, your husband/partner, you and your husband/partner jointly, or someone else?	1 Respondent<br>2 Husband/Partner<br>3 Respondent and Husband/Partner Jointly<br>4 Someone else (specify___)<br>98 DON’T KNOW<br>99 REFUSED TO ANSWER	

19	Who usually makes decisions about what food should be cooked each day? You, your husband/partner, you and your husband/partner jointly, or someone else?	1 Respondent<br>2 Husband/Partner<br>3 Respondent and Husband/Partner Jointly<br>4 Someone else (specify___)<br>98 DON'T KNOW<br>99 REFUSED TO ANSWER	
20	Has your partner accompanied you to an antenatal care or post-partum care appointment since you were enrolled in the study?	1 Yes<br>2 No<br>98 Don't know<br>99 Refused to answer	

**Section C. Health and sexual behavior**			
NO.	**QUESTIONS**	**CODING CATEGORIES**	
Now I have some questions about sexual activity, in order to gain a better understanding of some important aspects of your life.			
1	How old were you when you had sex by choice for the very first time?	AGE IN YEARS<br><br>ENTER:<br>"97" IF DON'T KNOW AGE OF FIRST SEX, BUT KNOW IT WAS WHEN FIRST STARTED LIVING WITH FIRST (HUSBAND OR PARTNER)<br>"98" DON'T KNOW<br>"99" REFUSE TO ANSWER	
2	In your lifetime, how many people have you had sex with?	(NUMBER)<br>98 DON'T KNOW<br>99 REFUSED TO ANSWER	
	For the next few questions, I will be asking you about your primary sexual partner. This could be a husband or someone with whom you have a committed relationship. This is not someone with whom you have only casual sex or sex in exchange for money or gifts.		
3	Do you currently have a primary sexual partner?	1 YES<br>2 NO<br>98 DON'T KNOW<br>99 REFUSED TO ANSWER	
4	What is your relationship to your primary sexual partner?	1 HUSBAND<br>2 LIVE-IN PARTNER<br>3 BOYFRIEND NOT LIVING WITH RESPONDENT<br>4 CASUAL ACQUAINTANCE<br>5 OTHER<br>98 DON'T KNOW<br>99 REFUSED TO ANSWER	
5	Has your primary partner ever had an HIV test?	1 YES<br>2 NO→8<br>98 Don't know→8<br>99 REFUSED TO ANSWER	
6	When was the most recent time your primary partner had an HIV test?	MM/YY<br><br>(98/98=Don't Know)<br>99 REFUSED TO ANSWER	
7	Do you know your primary partner's HIV status?	1 Yes<br>2 No →10<br>98 Unknown→10<br>99 REFUSED TO ANSWER	
8	Primary partner's status	1 Positive<br>2 Negative<br>99 REFUSED TO ANSWER	If know in Q7
9	How long have you known your primary partner's HIV status?	1 <1 month<br>2 1-6 months<br>3 >6 months<br>98 Don't Know	If know in Q7

		99 Refused	
10	In the past 6 months, have you discussed the need for your primary partner to go for an HIV test?	1 Yes<br>2 No<br>98 DON'T KNOW<br>99 REFUSED TO ANSWER	
11	Do you think that chances that your primary partner currently has HIV/AIDS are high, moderate, low, or do you think you are at no risk at all?	1 High<br>2 Moderate<br>3 Low<br>4 No risk at all<br>98 DON'T KNOW<br>99 REFUSED TO ANSWER	
12	How often do you use condoms when you have sex with your primary partner?	1 None of the time<br>2 Some of the time<br>3 Most of the time<br>4 All of the time<br>98 DON'T KNOW<br>99 REFUSED TO ANSWER	
13	Has there ever been a time when you wanted to use a condom, your primary partner refused, and you still had sex?	1 Yes<br>2 No<br>98 DON'T KNOW<br>99 No answer	
14	When was the <u>most recent</u> time you had sexual intercourse?	1 Past 7 days<br>2 Past month<br>3 Past 3 months<br>4 Past 6 months<br>5 Past 1 year<br>6 More than 1 year ago<br>98 DON'T KNOW<br>99 REFUSE TO ANSWER	
15	The last time you had sexual intercourse, did you use a condom?	1 YES<br>2 NO<br>98 DON'T KNOW<br>99 REFUSED TO ANSWER	
16	Have you had sexual intercourse with any person who is not your primary sexual partner in the last 12 months?	1 YES<br>2 NO →18<br>98 DON'T KNOW<br>99 REFUSED TO ANSWER	
17	In total, with how many different people have you had sexual intercourse in the past <u>12 months</u>?<br><br>IF NON-NUMERIC RESPONSE, PROBE TO GET AN ESTIMATE	NUMBER OF PARTNERS<br><br>98 DON'T KNOW<br>99 REFUSE TO ANSWER<br>IF NUMBER OF PARTNERS IS 97 OR MORE, WRITE '97'.	

**Section E. HIV Testing**			
**NO.**	**QUESTIONS**	**CODING CATEGORIES**	
1	Have you ever been tested for HIV?	1 Yes<br>2 No→<br>98 DON'T KNOW<br>99 REFUSED TO ANSWER	
2	If yes, what type of HIV testing have you had before? (check all that apply)	__ Individual HIV test at a VCT centre<br>__ Individual HIV test at community-based testing<br>__ Couples based HIV testing at a VCT centre<br>__ Couples based HIV testing at community-based testing<br>__ HIV self-test<br>__ Other (specify)	
3	How many times have you been tested for HIV in the past 12 months?	NUMBER OF TESTS (2 DIGITS)<br>0 is not allowed<br>98 DON"T KNOW<br>99 REFUSED TO ANSWER	

4	When was the most recent time you were ever tested for HIV?	1 ≤ 3 MONTHS AGO<br>2 3-6 MONTHS AGO<br>3 6-12 MONTHS AGO<br>4 12-23 MONTHS AGO<br>5 2 YEARS OR MORE AGO<br>98 DON’T KNOW<br>99 REFUSED TO ANSWER	
5	What was the result of your most recent HIV test?	1 HIV-positive →go to question 9<br>2 HIV-negative<br>3 Indeterminate<br>98 Don’t Know<br>99 REFUSED TO ANSWER	
6	Do you think your chances of having or acquiring HIV/AIDS are high, moderate, low, or no risk at all?	1 HIGH<br>2 MODERATE<br>3 LOW<br>4 NO RISK AT ALL<br>98 DON’T KNOW<br>99 REFUSED TO ANSWER	
7	Why do you think that you have a <u>low</u> chance or <u>no risk</u> of having or acquiring AIDS?<br><br>SELECT ALL MENTIONED	1 IS NOT HAVING SEX<br>2 USES CONDOM<br>3 HAS ONLY ONE PARTNER<br>4 LIMITS THE NUMBER OF PARTNERS<br>5 PARTNER HAS NO OTHER PARTNERS<br>6 OTHER_______________ (SPECIFY)<br>98 DON’T KNOW<br>99 REFUSED TO ANSWER	If Q6=3 or 4
8	Why do you think that you have a <u>moderate</u> or <u>high chance</u> of having or acquiring AIDS?<br><br>SELECT ALL MENTIONED	1 DOES NOT USE CONDOM<br>2 HAS MORE THAN ONE SEX PARTNER<br>3 HAS SEX IN EXCHANGE FOR MONEY OR GOODS<br>4 HAD BLOOD TRANSFUSION/INJECTION<br>5 OTHER________________ (SPECIFY)<br>98 DON’T KNOW<br>99 REFUSED TO ANSWER	If Q6=1 or 2
9	Had you ever heard of HIV self-testing before today?	1 YES<br>2 NO →12<br>98 DON’T KNOW<br>99 REFUSED TO ANSWER	
10	Have you ever used an HIV self-test before today?	1 YES<br>2 NO<br>98 DON’T KNOW<br>99 REFUSED TO ANSWER	If Q9=1
11	What was the result of your most recent HIV self-test?	1 HIV-positive<br>2 HIV-negative<br>3 Indeterminate<br>99 REFUSED TO ANSWER	If Q10=1
12	How comfortable do you feel about asking your primary sexual partner to use an HIV self-test?	1 VERY COMFORTABLE<br>2 SOMEWHAT COMFORTABLE<br>3 NEUTRAL<br>4 SOMEWHAT UNCOMFORTABLE<br>5VERY UNCOMFORTABLE<br>98 DON’T KNOW<br>99 REFUSED TO ANSWER	

Section D. Gender-Based Violence

Interviewer: "I always ask the following questions because some people are in relationships where they don't feel safe and this affects their health."			
1	If you told your partner that you came here for health services today, would he react angrily or negatively?	1 YES<br>2 NO<br>98 Don't Know<br>99 Refused	
	In the past 12 months, has your partner…		
2	Pushed, grabbed, slapped, choked, hit or kicked you?	1 YES<br>2 NO<br>98 Don't Know<br>99 Refused	
3	Threatened to hurt you, your children or someone close to you?	1 YES<br>2 NO<br>98 Don't Know<br>99 Refused	
4	Insulted you or made you feel bad about yourself?	1 YES<br>2 NO<br>98 Don't Know<br>99 Refused	
5	Taken away money or resources that you/your children need to survive?	1 YES<br>2 NO<br>98 Don't Know<br>99 Refused	
6	Sent you back to your family home?	1 YES<br>2 NO<br>98 Don't Know<br>99 Refused	
7	Forced you to have sex when you did not want to?	1 YES<br>2 NO<br>98 Don't Know<br>99 Refused	
8	Has your partner tried to get you pregnant when you didn't want to be?	1 YES<br>2 NO<br>98 Don't Know<br>99 Refused	
9	If you wanted to use a condom or another family planning method, would you be afraid to ask your partner?	1 YES<br>2 NO<br>98 Don't Know<br>99 Refused	
10	Are you worried that your partner will be angry and/or hurt you if he finds out you were tested for HIV?	1 YES<br>2 NO<br>98 Don't Know<br>99 Refused	
11	Do you feel unsafe returning to your home today?	1 YES<br>2 NO<br>98 Don't Know<br>99 Refused	

REVIEW HIV SELF TESTING MATERIALS AND COUNSELING

**Section F: Interviewer's Observations**<br><br>**TO BE ANSWERED BY INTERVIEWER AFTER INTERVIEW IS FINISHED**			
NO.	QUESTIONS	CODING CATEGORIES	
1	WHO ELSE WAS PRESENT DURING THIS INTERVIEW?	1 NO ONE<br>2 A CHILD 5 YEARS OLD OR LESS	

	SELECT ALL THAT APPLY	3 A CHILD OLDER THAN 5 YEARS<br>4 HUSBAND OR PARTNER<br>5 AN ADULT, MEMBER OF THE HOUSEHOLD WHO IS NOT RESPONDENT'S PARTNER<br>6 AN ADULT, NOT MEMBER OF THE HOUSEHOLD	
2	WHAT IS YOUR EVALUATION ABOUT THE SERIOUSNESS AND ATTENTION OF THE RESPONDENT?	1 EXCELLENT<br>2 GOOD<br>3 MEDIUM<br>4 BAD<br>5 VERY BAD	
3	Interviewer: Use this field to record reasons for selecting options 4 and 5 under #3 above, and any special comments/info/problems		

**END TIME OF INTERVIEW**<br>(HH:MM)
